# Placental Teratoma Presenting as a Lobulated Mass behind the Neck of Fetus: A Case Report

**DOI:** 10.1155/2012/857230

**Published:** 2012-06-19

**Authors:** Adiga Prashanth, Rai Lavanya, K. M. Girisha, Anjali Mundkur

**Affiliations:** ^1^Department of Obstetrics and Gynaecology, Kasturba Medical College, Manipal University, Manipal 576104, India; ^2^Genetics Clinic, Department of Pediatrics, Kasturba Medical College, Manipal University, Manipal 576104, India

## Abstract

Placental teratoma is a rare nontrophoblastic benign tumour, which is thought to arise from germ cells. These tumours contain elements derived from multiple germ cell layers. We report a case of teratoma, where on ultrasound; there were two echogenic masses of 4 cm × 5 cm and 3 cm × 4 cm, arising from the placenta. Elective lower segment cesarean section was done in view of breech presentation at 38 weeks of gestation. Gross examination of the placenta showed two lobulated masses of 5 cm × 5 cm and 4 cm × 4.5 cm, respectively. Histopathological examination of the placenta was suggestive of teratoma of the placenta. The fetus was normal.The maternal and fetal outcome was good.

## 1. Introduction 

Teratoma of the placenta is a rare nontrophoblastic benign tumour. Placental teratomas are thought to arise from germ cells, which migrate from the dorsal wall of the yolk sac. These tumours contain elements derived from multiple germ cell layers. We report a case of teratoma in a twenty-five- year-old lady posing diagnostic challenges.

## 2. Case Report

**Figure 1:**
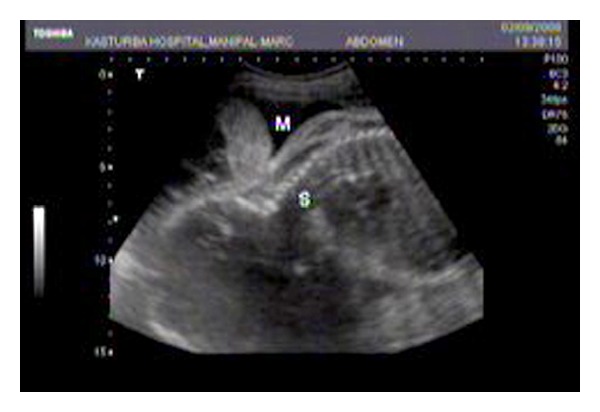
Showing an echogenic mass in close proximity to the occipital region of the fetus.

**Figure 2:**
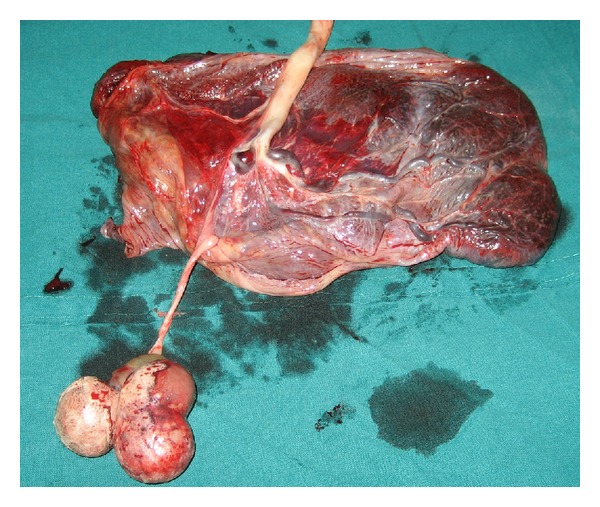
Showing the placenta after delivery with the presence of two masses attached to the placenta.

A twenty-five-year-old second gravida at 36 weeks of gestation was referred to our institution as a case of encephalocele with preeclampsia complicating pregnancy, for further management. The patient had regular antenatal visits and was diagnosed to have preeclampsia at 32 weeks of gestation. She was not on any medication. A target scan done at 19 weeks was said to be normal. On examination, her blood pressure was 140/90 mmHg. Systemic examination was normal. Obstetric examination revealed a single live fetus corresponding to thirty-six weeks in breech presentation. On ultrasound examination (Toshiba Nemio 10, curvilinear, 5 MHz probe), there was a fetus corresponding to 36 weeks in breech presentation with an amniotic fluid index of 14. There were two echogenic masses of 4 cm × 5 cm and 3 cm × 4 cm, arising from the placenta in close proximity with the occiput of the fetus (Figure 1). One was cystic, and the other showed lobulation/septation. As there was no defect in the skull, encephalocele was considered to be less likely. Their attachment to other structures could not be traced. Gross fetal intracranial anatomy was normal. On Doppler ultrasound examination, there was minimal vascularity on the surface of the mass. Although magnetic resonance imagining (MRI) is the investigation of choice when an encephalocele is suspected, this investigation could not be done due to patients financial constraints. At thirty-eight weeks of gestation, the patient was posted for cesarean section for breech presentation, and a live male baby of 2.8 kg was delivered. There were no gross external deformities or anomalies in the neonate. The placenta measured 15 cm × 12 cm × 4 cm and weighed 250 grams. Maternal and fetal surfaces of the placenta were unremarkable. There were two masses attached to the membranes measuring 5.5 cm × 5 cm and 4 cm × 3 cm, respectively (Figure 2). The first mass appeared lobulated. The second mass appeared cystic. Histopathological examination of the placenta was unremarkable. Histopathological examination of the masses showed lining of epidermis with attached adnexa of varying maturity. There were lobules of adipose tissue, stromal myxoid changes and islands of osteoid, and cartilage and smooth muscles tissues. The above findings were suggestive of benign teratoma of the placenta. The patient had uneventful postoperative period and was discharged on the seventh postoperative day. Neonate also did not have any problems.

## 3. Discussion

The first case report of placental teratoma was by Morville in the year 1925 [1]. Placental teratomas always lie between the amnion and the chorion, usually on the fetal surface of the placenta, but sometimes, within the membranes [2]. The origin of placental teratoma is obscure. Placental teratomas originate from the abnormal migration of embryonic germ cells [3]. These cells migrate through the umbilical cord before arriving in the placenta [4]. The prenatal diagnosis of placental teratoma is based on the presence of tissues of varied echogenicity such as fat, calcification, and fluid on ultrasound [5]. The present case was different from the other cases in that the echogenicity of the masses was the same as the placenta, and the teratoma was seen as an outpouching from the placenta. The differential diagnosis of placental teratoma is a fetus amorphous, which is a blighted fetus arising from a multiple pregnancy. The distinguishing feature in fetus amorphous is the presence of some growth organization with central skeletal development, and with partial or complete formation of a vertebral column. Second differentiating feature is a separate, poorly developed umbilical cord which is either attached to the placenta or to its twin, or to a separate placenta.

In the present case, the location of the mass near the occiput suggested the possibility of an encephalocele. Though the absence of a skull defect an encephalocele is less likely, a small defect could not be confidently ruled out during sonographic evaluation. Moreover, the thin attachment to the membranes gave the impression of a free floating mass near the nape of the fetus in the uterine cavity.

To conclude, placental teratoma is a benign nontrophoblastic tumour which has a favorable prognosis. During the antenatal sonogram, however, it may pose diagnostic challenges. 

## References

[1] Morville P (1925). Une teratoma placentaire. *Obstetrics & Gynecology*.

[2] Elagöz S, Aker H, Cetin A (1998). Placental teratoma—a case report. *European Journal of Obstetrics Gynecology and Reproductive Biology*.

[3] Shimojo H, Itoh N, Shigematsu H, Yamazaki T (1996). Mature teratoma of the placenta. *Pathology International*.

[4] Fox H (1995). *Pathology of the Placenta*.

[5] Ahmed N, Kale V, Thakkar H, Hanchate V, Dhargalkar P (2004). Sonographic diagnosis of placental teratoma. *Journal of Clinical Ultrasound*.

